# Survival outcomes in patients with *de novo* metastatic Merkel cell carcinoma according to site of metastases

**DOI:** 10.3389/fonc.2024.1444590

**Published:** 2024-09-16

**Authors:** Karam Khaddour, Mofei Liu, Emily Y. Kim, Furkan Bahar, Matheus M. Lôbo, Anita Giobbie-Hurder, Ann W. Silk, Manisha Thakuria

**Affiliations:** ^1^ Department of Medical Oncology, Dana-Farber Cancer Institute, Boston, MA, United States; ^2^ Merkel Cell Carcinoma Center of Excellence, Dana-Farber Cancer Institute, Boston, MA, United States; ^3^ Harvard Medical School, Boston, MA, United States; ^4^ Department of Data Science, Division of Biostatistics, Dana-Farber Cancer Institute, Boston, MA, United States; ^5^ Skin Cancer Department, A.C. Camargo Cancer Center, São Paulo, SP, Brazil; ^6^ Department of Dermatology, Brigham and Women’s Hospital, Boston, MA, United States

**Keywords:** Merkel cell cancer, metastases, *de novo*, survival, outcome, organ sites of metastasis, non melanoma skin cancer

## Abstract

**Introduction:**

Merkel cell carcinoma (MCC) is a rare and aggressive neuroendocrine malignancy of the skin with a predilection for metastases. This study investigates the clinical outcomes in patients presenting with *de novo* Stage IV MCC according to the metastatic site(s) at presentation.

**Materials and methods:**

Patients who presented with one or more sites of distant metastatic MCC at initial diagnosis between 2009 and 2023 were identified. The presence or absence of one or more metastases in each organ was categorized for each patient at the time of diagnosis. Overall survival (OS) and progression-free survival (PFS) were estimated using the Kaplan-Meier method. Competing risk analysis was used to estimate the cumulative occurrence risk of MCC-specific death. Fisher’s exact test was used for response rate analysis. Results were considered statically significant if *p* < 0.05.

**Results:**

Thirty-four patients presented with *de novo* distant metastatic MCC. There was no association between the number of metastatic sites at diagnosis and OS (*p*= 0.58), PFS (*p*=0.79), or response rates (*p*=0.53). However, the presence of bone metastases was associated with significantly shorter OS (8.2 versus 25.2 months, HR: 2.4, 95% CI 1.01-5.7, *p*= 0.04). MCC-specific death in patients with lymph node metastases was significantly lower than in patients without (HR: 0.28, 95% CI: 0.09-0.87, *p*= 0.013). The presence of bone metastases tended to associate with an increased risk of MCC-specific death, although not statistically significant. The location of metastases was not associated with the response rate to first-line treatment. There was no significant association between site of metastases and PFS.

**Conclusion:**

In this cohort of patients with *de novo* metastatic MCC, the presence of bone metastases, but not the number of organs involved, was associated with significantly worse OS. The presence of lymph node metastases was associated with lower MCC-specific death. Further research is warranted in larger cohorts to investigate the impact of the location of metastases on clinical outcomes.

## Introduction

Merkel cell carcinoma (MCC) is a rare neuroendocrine skin cancer. It represents less than 1% of skin cancers and has an annual incidence of approximately 3000 cases per year in the United States (1, 2). The estimated overall survival (OS) at 2 years is 30% for patients with Stage IV disease (3). Due to the low incidence of MCC, there is a lack of understanding if the number of metastases or site of metastases impacts clinical outcomes in patients with stage IV disease (4). A retrospective study by Lewis, et al, described the relationship between sites of metastases and prognosis in 215 patients with MCC (5). The majority of the patients included in that analysis did not present with metastatic disease, but later developed metastatic disease during surveillance. The authors reported that liver metastases were associated with a higher risk of death due to MCC compared to skin and body wall metastases (HR:2.13, 95% CI 1.00-4.55, *p* = 0.05). Recently, we reported on the patterns of metastatic recurrence in 151 patients with loco-regional MCC treated at Dana-Farber/Brigham and Women’s Hospital (DF/BWH) who were found to have metastatic disease during surveillance (6). We, too, found that the presence of liver metastases was associated with shorter OS; additionally, we found that the presence of bone metastases was associated with shorter OS. MCC tumors that tend to present as *de novo* MCC could be biologically distinct from MCCs that initially present with locoregional disease. For example, they could be more aggressive, and the sites of disease would not be impacted by initial therapies such as surgery and radiation therapy. Herein, we report on the prognostic impact associated with the location of metastases in patients diagnosed with *de novo* stage IV MCC at DF/BWH.

## Materials and methods

This study included patients with a confirmed histological diagnosis of MCC who consented to Dana-Farber/Harvard Cancer Center Protocol #09-156, and who had one or more distant metastases at the time of diagnosis (*de novo* MCC). We collected data using the Research Electronic Data Capture (REDCap), which was cross-checked through review of patients’ electronic medical records by a medical oncologist. Data that was collected and used for analysis included: age at diagnosis, gender, race, primary skin location (when applicable), metastatic site(s) at the time of diagnosis, date of diagnosis, first-line treatment, type of cancer-directed treatment, best response on imaging, date and location of progression, imaging modality at the time of diagnostic staging, antibodies to the MCC oncoprotein when available (AMERK), vital status, cause of death, and last follow up information (data cutoff date 12/31/2023).

### Statistical analysis

Patients might have multiple metastatic sites and each metastatic site was treated as a binary variable (metastasis or multiple metastases in that organ site was captured as present/absent). Response assessment on imaging was categorized as complete response, partial response, stable disease, or progressive disease as annotated by a radiology report, among which, patients with complete and partial response were considered as responder, otherwise non-responder. Fisher’s exact test was used for response rate analysis and results were considered statistically significant if *p* < 0.05. Overall survival (OS) was defined as months between date of initial diagnosis of metastatic MCC, and date of death from any cause, or censored at the date of last follow up. Progression-free survival (PFS) was defined as months between date of initial diagnostic biopsy and date of progression/death or censored at the date of last follow up, whichever occurred first. Kaplan-Meier method was used to estimate survival, and Cox Proportional Hazard model was used to calculate hazard ratio. Log-rank test was used to compare the survival between groups. MCC-specific death (MSD) was analyzed using univariate competing risks regression with subdistribution hazard, which represents the MSD rate per month as well as the influence of competing events, defined as death caused by any other reasons except MCC (7, 8). Fine-Gray method was used to estimates the instantaneous rate of occurrence of the MCC-specific death in subjects who have not yet experienced an event of that type. Cumulative incidence of the MSD was provided at 1, 3 and 5 years. Comparison of MSD between two groups was assessed with Grey’s test (9).

## Results

Between 2009 and 2023, 35 patients with *de novo* MCC were identified. One patient was excluded from analysis because the patient had no response or survival information available, resulting in an analytic cohort of 34 patients. Baseline characteristics of the study cohort are described in **Table 1**. The most frequently involved metastatic site at diagnosis was distant lymph nodes, followed by bone, liver, lung, and soft tissue, respectively. The presence of a primary skin lesion was identified in 41.2% of patients (n= 14), and 58.8% presented without a known primary skin lesion. Positron emission tomography/computed tomography (PET/CT) scan was performed for diagnostic staging in 85% of patients, and brain imaging was performed in 38% of patients at the time of diagnosis. There were 16 patients (37%) who had AMERK serology testing available, of whom 9 (56%) were AMERK-positive. Regarding first-line treatment, 32.4% received immune checkpoint inhibitors (ICI), and 58.8% received chemotherapy. The median follow-up of these patients was 4.8 years.

**Table 1:** Baseline characteristics of patients with *de novo* metastatic Merkel cell carcinoma.

	N=34
Age	
Mean (SD)	69.9 (10.3)
Median [Min, Max]	70.2 [39.4, 90.8]
Gender	
Female	14 (41.2%)
Race	
White	30 (88.2%)
Black or African American	2 (5.9%)
Hispanic	1 (2.9%)
**Primary Skin Lesion**	14 (41.2%)
Site of Distant Metastases	
Lymph Node Metastases	28 (82.4%)
Bone Metastases	10 (29.4%)
Soft Tissue Metastases	7 (20.6%)
Liver Metastases	7 (20.6%)
Lung Metastases	7 (20.6%)
Brain Metastases	3 (8.8%)
Pancreas Metastases	2 (5.9%)
Skin or Subcutaneous Metastases	2 (5.9%)
Adrenal Metastases	1 (2.9%)
Kidney Metastases	1 (2.9%)
Number of sites of metastases	
1	14 (41.2%)
2	10 (29.4%)
3	5 (14.7%)
4	5 (14.7%)
Best Response to Palliative First Line Systemic Therapy	
Complete Response	11 (32.4%)
Partial Response	9 (26.5%)
Stable Disease	3 (8.8%)
Progressive Disease	9 (26.5%)
Missing	2 (5.9%)
First line treatment	
Immunotherapy	11 (32.4%)
Chemotherapy	20 (58.8%)
Other	3 (8.8%)

### Overall survival

**Figure 1:**
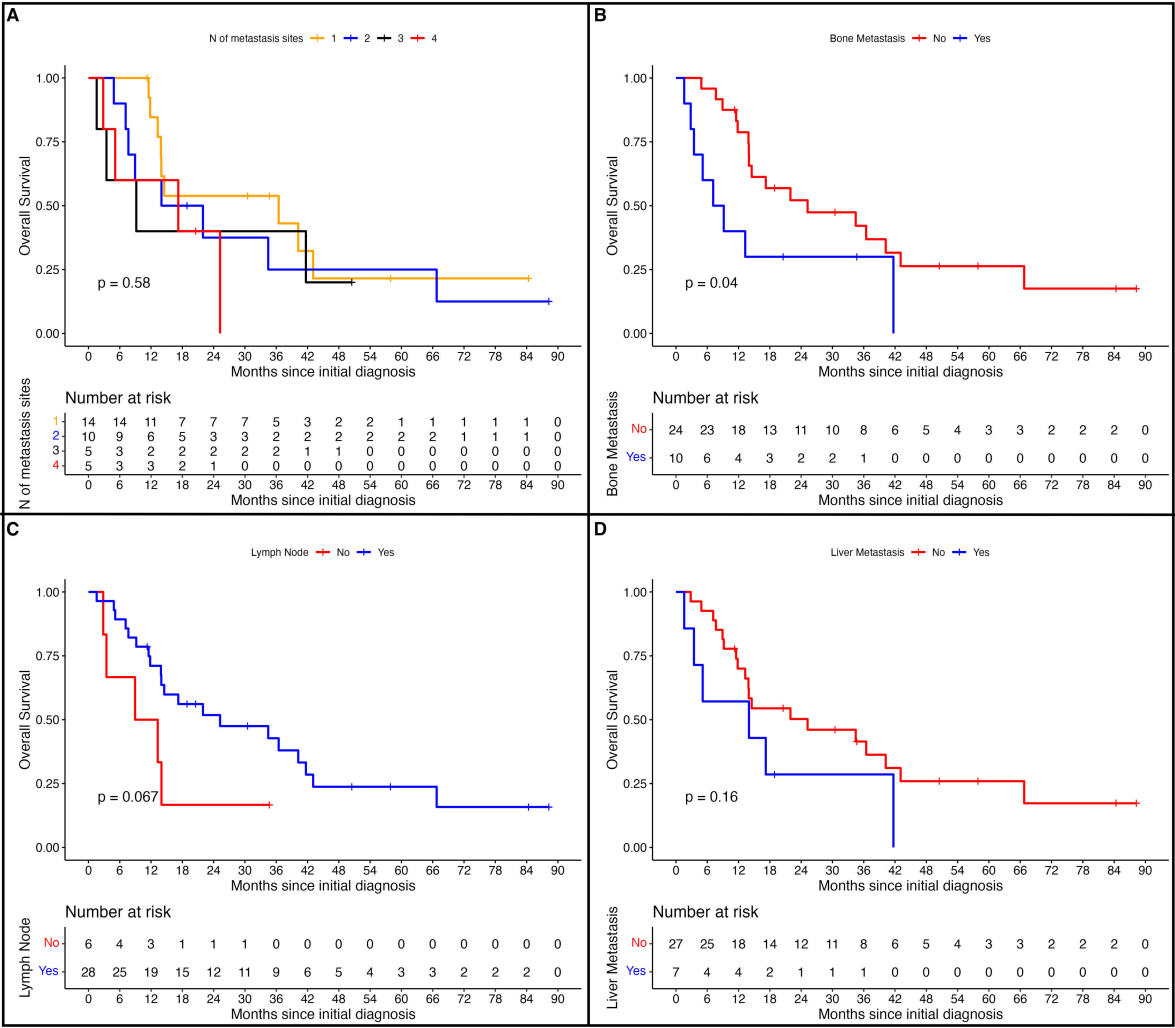
Overall survival in patients with *de novo* metastatic Merkel cell carcinoma. **(A)** overall survival in patients according to number of metastatic sites at diagnosis, **(B)** overall survival in patients with bone metastases, **(C)** overall survival in patients with lymph node metastases, and **(D)** overall survival in patients with liver metastases.

The median OS for the study cohort was 17.2 months (95% CI 13.9 – 41.7 months). Notably, there was no association between the number of metastatic sites at presentation and OS (*p*= 0.58) (**Figure 1A**). Among the various sites of metastatic disease, patients with bone metastases had significantly shorter OS than other sites (8.1 versus 25.2 months, HR:2.41, 95% CI 1.01-5.74, *p*= 0.04) (**Figure 1B**). The presence of lymph node metastases compared to other sites trended toward improved OS but was not statistically significant (25.2 versus 11.1 months; HR:0.4, 95% CI 0.14-1.1, *p*= 0.067) (**Figure 1C**). The presence of liver metastases compared with all other metastatic sites was not associated with different OS (*p*= 0.16) (**Figure 1D**).

### Merkel cell carcinoma-specific death

**Figure 2:**
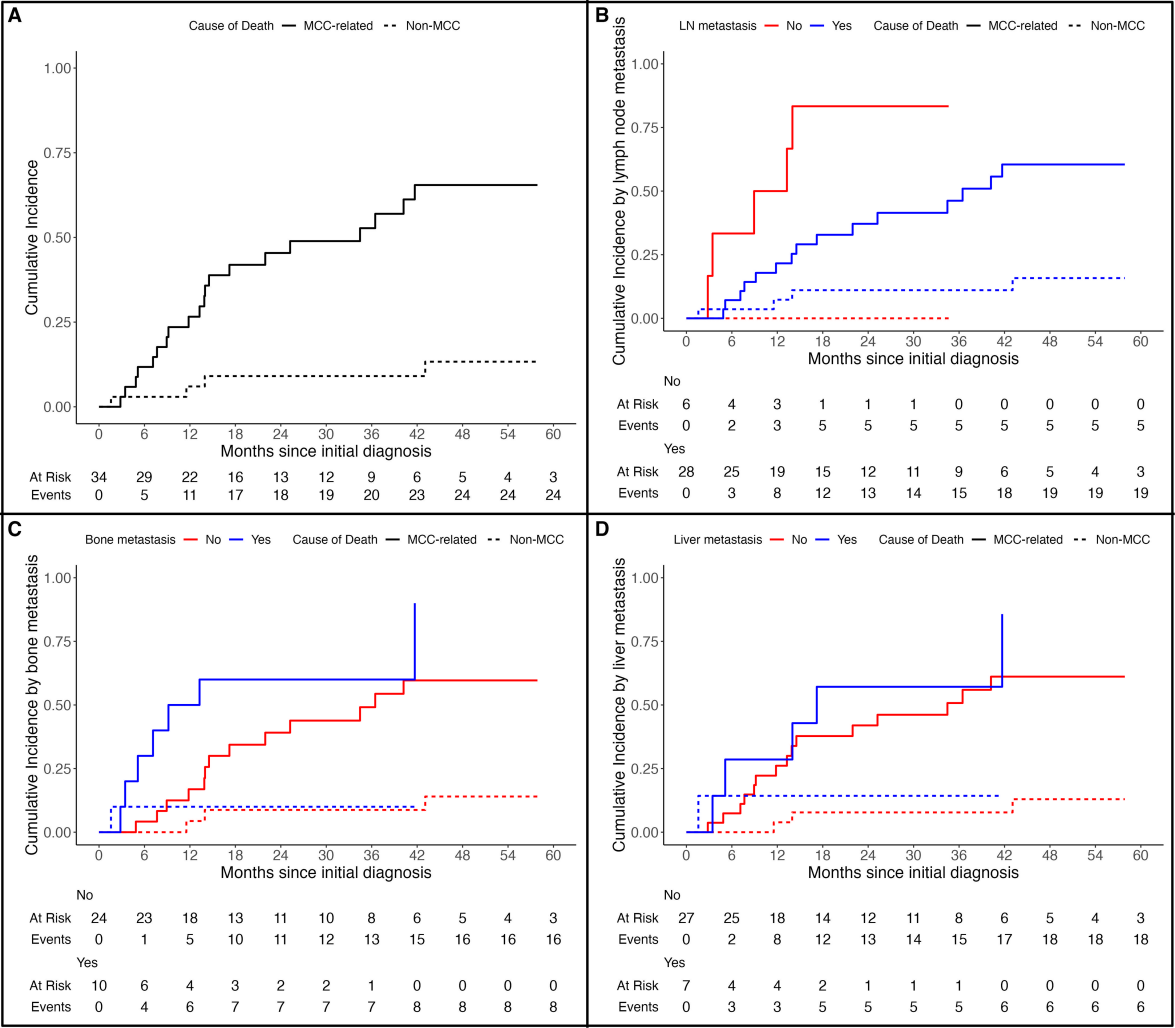
Cumulative incidence of Merkel cell carcinoma- specific death (MSD) in **(A)** all cohort, **(B)** according to presence of lymph node metastases, **(C)** according to presence of bone metastases, and **(D)** according to presence of liver metastases.

The 1-year, 3-year, and 5-year cumulative incidence of death due to MCC was 27% (95% CI: 13%-42%), 53% (95% CI: 33%-69%), and 65% (95% CI: 44%-80%) respectively (**Figure 2A**). Patients with lymph node metastases had a significantly lower risk of MCC-specific death (MSD) (HR:0.28, 95% CI: 0.09-0.87, *p*=0.013) (**Figure 2B**). Bone metastases were likely associated with risk of MSD, although the difference was not statistically significant (HR:2.06, 95% CI: 0.81-5.26, *p*= 0.087) (**Figure 2C**). Liver metastases were not statistically associated with the risk of MSD (HR:1.52, 95% CI: 0.60-3.83, *p*= 0.4) (**Figure 2D**).

### Progression-free survival and response rates

The median PFS in the cohort was 7.8 months (95% CI: 5.98-13.4 months). There was no association between the number of metastatic sites at presentation and PFS (*p*= 0.79). The presence of bone metastases was not associated with worse PFS (*p*= 0.41). Similarly, the presence of liver metastases, liver and bone metastases or lymph nodes was not associated with worse PFS. There were too few patients to investigate PFS according to treatment type. Neither the number of metastatic sites nor the specific site involved was associated with response rate to first line treatment for *de novo* metastatic MCC (*p*= 0.53).

## Discussion

In patients with stage IV MCC, risk stratification is not well characterized. Recently, we showed that patients diagnosed with stage I-III MCC who subsequently experienced disease recurrence or progression with distant metastases had shorter OS if they had liver or bone involvement at the time of recurrence (6). In the current study, we identify patterns of distant metastases in patients presenting with *de novo* stage IV MCC. The presence of bone lesions, but not the number of organs involved, was associated with shorter OS. We investigated the impact of metastatic organ involvement on MSD given competing causes of death in an elderly population. The presence of bone metastases or liver metastases trended numerically towards increased risk of MSD, and lymph node involvement was associated with significant decreased MSD. The most common location of metastases was the distant lymph nodes, consistent with prior studies demonstrating the propensity of MCC for lymphatic spread (10). Other commonly involved sites in our cohort included the liver, soft tissue, and bone. Unlike in melanoma, the presence of brain metastases at diagnosis was low (~ 9%), consistent with previous institutional reports (11, 12). In contrast to patients who presented with local or regional MCC in our previous report, *de novo* metastatic patients presenting with liver metastases did not have shorter OS (6). In two large cohort studies of patients with MCC from the Surveillance, Epidemiology, and End Result (SEER) database, the frequency of liver, and bone metastases was (26-39%) and (20-28%) respectively (13, 14). The SEER database analysis revealed worse OS in patients with liver and bone metastases, but the site of metastases was not associated with MSD (13, 14). Despite the large sample size in these prior studies compared to our study, inaccurate data capture, unrecorded variables, and coding variation from the SEER database could limit the interpretation of the results (15). As such, data that overcomes these limitations with detailed and accurate identification of metastatic patterns and their prognostic impact on survival could provide insight on risk stratification (5, 6, 16).

Organ-specific metastases can have a predictive value in different tumor types (17). Several studies evaluating sites of metastases in the era of immunotherapy suggest that specific organ involvement could lead to different response and survival patterns (18–21). For example, the presence of liver and brain metastases was associated with shorter OS in patients with melanoma and non-small cell lung cancer treated with immunotherapy (21, 22). In another study, the presence of bone metastases in patients with renal cancer predicted worse outcomes to immunotherapy (18). Similarly, bone metastases in patients with melanoma are associated with worse survival (23, 24). These findings are important as immune checkpoint inhibitors constitute the mainstay of treatment in patients with metastatic MCC (25, 26). In our cohort, despite the shorter OS in patients with bone metastasis, there was no difference in response rate to treatment. However, the interpretation of this finding is difficult given the small number of patients and the low proportion of patients receiving immunotherapy as a first-line treatment (32%).

The worse survival outcomes in various cancer types associated with bone metastases could suggest a distinct tumor microenvironment (TME) that is more immunosuppressive and leads to a more aggressive disease pattern (27, 28). The potential suppressive TME of bone metastases has been investigated in patients with prostate cancer who were found to have low response rates to immunotherapy and worse survival (27). In this study, resistance to immunotherapy was mediated by osteoclastic activity and an increase in TGF-B, leading to inhibition of Th1 subsets (27).

Limitations to our study include the retrospective nature, the lack of uniform imaging, and the small sample size. Although a minority of patients had brain imaging, brain imaging is not recommended at diagnosis unless prompted by neurologic symptoms (12).

In conclusion, we report on the first pure cohort of *de novo* metastatic disease and their clinical outcomes according to the site of distant metastasis at diagnosis. The presence of bone metastases, but not the number of overall organs involved, was associated with significantly worse overall survival. If bone involvement is confirmed to be a negative prognostic factor in an independent dataset, it would warrant fundamental investigation to analyze the interplay between bone microenvironment and metastatic MCC.

## Data Availability

The raw data supporting the conclusion of this article may be made available by the authors upon request and per regulations and procedures at the Dana- Farber/ Harvard Cancer Center. Further inquiries can be directed to the corresponding author.
